# The impact of sewage sludge on the fungal communities in the rhizosphere and roots of barley and on barley yield

**DOI:** 10.1515/biol-2021-0024

**Published:** 2021-03-05

**Authors:** Katarína Ondreičková, Michaela Piliarová, Lenka Klčová, Alžbeta Žofajová, Jozef Gubiš, Miroslav Horník, Marcela Gubišová, Martina Hudcovicová, Ján Kraic

**Affiliations:** Department of Applied Biology and Genetics, National Agricultural and Food Centre – Research Institute of Plant Production, Bratislavská cesta 122, 921 68, Piešťany, Slovak Republic; Department of Biotechnologies, Faculty of Natural Sciences, University of Ss. Cyril and Methodius, Námestie J. Herdu 2, 917 01, Trnava, Slovak Republic; Department of Ecochemistry and Radioecology, Faculty of Natural Sciences, University of Ss. Cyril and Methodius, Námestie J. Herdu 2, 917 01, Trnava, Slovak Republic

**Keywords:** alpha diversity, arbuscular mycorrhizal fungi, barley, fungal diversity, sewage sludge

## Abstract

Current problems with sewage sludge (SS) disposal could be solved by application to agricultural land considering its fertilizer properties and ability to improve soil condition. However, SS may contain heavy metals as well as pathogenic microorganisms. In this study, molecular analysis of partial 18S rRNA gene was used to study the impact of SS application into the soil on the genetic diversity of fungal communities, especially arbuscular mycorrhizal fungi in the rhizosphere and roots of barley. These samples were collected on three dates from the control soil without SS and from the soil with the addition of SS at the concentrations of 5 and 15 t ha^−1^. Fungal alpha diversity in the rhizosphere of barley was affected by SS differently than in barley roots. In addition, principal component analysis and cluster analysis revealed that fungal communities were strongly influenced by the SS addition into the soil, sample type, and the sampling date. This approach was complemented by an evaluation of the basic parameters of barley production and the response of these parameters to the presence of SS in the soil. The plant height increased with increasing SS concentration and the thousand seed weight significantly increased at the concentration of 5 t ha^−1^ SS but significantly decreased in 15 t ha^−1^.

## 1 Introduction

Globally, the amount of sewage sludge (SS) as a byproduct of the wastewater treatment process has been constantly increasing. Therefore, there is a serious problem with its disposal. On the contrary, there are several ways to dispose SS in the European Union (EU). Available data from Eurostat about the disposal of SS in the EU in 2018 indicate that 20.2% was applied to agricultural soil, 16.9% was composted, 13.7% was landfilled, 26.7% was incinerated, and 22.6% was disposed of through other means [1]. However, it should be noted that not all EU countries have provided data on SS disposal and therefore are not included in these statistics. In the Slovak Republic, the situation is different from this European average. In 2018, 45.5% of SS was composted, 20.2% was landfilled, 20.9% was incinerated, and 13.5% was used in other applications. In contrast to the EU, where the most SS was applied to agricultural soil, in the Slovak Republic it was 0%, and this trend remains from 2014 to the present [1]. The advantage of the application of SS to agricultural soil is the content of plant micro- and macronutrients and organic matter [2], which makes it is valuable for its fertilizing and soil conditioning properties [3]. On the contrary, SS may be a source of chemical (heavy metals) and biological contamination (thermo-tolerant coliform bacteria, fecal streptococci, and others) [3,4,5]. The concentrations of heavy metals, as well as pathogenic microorganisms, can simultaneously limit the acceptability for the application of SS to agricultural land. Therefore, its usage by farmers in the EU is defined by Council Directive 86/278/EEC of 12 June 1986 on the protection of the environment, particularly of the soil when SS is used in agriculture [6]. In 2014, this directive was evaluated in Final Report [7] which also evaluated four other waste stream directives and subsequently amended by Decision (EU) 2018/853 [8]. Certain SS materials (e.g., precipitated phosphate salts or materials exclusively obtained through the thermochemical conversion under non-oxygen and oxygen-limiting conditions [9]) may be part of the EU fertilizing products as defined by Regulation (EU) 2019/1009 of the European Parliament and the Council of 5 June 2019 [10]. This new and revised form of EU Regulation (EC) No 2003/2003 [11] extends the previous scope to secondary-raw-material-based fertilizing products. Furthermore, the European Commission describes the introduction of further measures to reduce waste and ensure that the EU has a well-functioning internal market for high-quality secondary raw materials [12]. At the same time, this Circular Economy Action Plan [12] is a part of the European Green Deal [13] that provides a roadmap for action to promote resource efficiency through a transition to a clean circular economy as well as biodiversity restoration and pollution reduction. These measures, as well as other measures not mentioned here, have been introduced by the European Commission to protect, preserve, and improve the environment in Europe for present and future generations.

As already mentioned, SS application to agricultural land appears to be a suitable solution considering its fertilizing properties and ability to improve the soil’s physical, chemical, and biological conditions [2,3,14]. Thus, sludge alters soil properties, which can subsequently affect soil microorganisms as well as plants. In general, better-quality soil usually has high microbial biomass content and enzyme activity, and so soil microorganisms can be used as indicators of soil quality [15]. Soil microorganisms play a crucial role in various biogeochemical cycles, as well as in the formation of soil structure, the decomposition of soil organic matter, and the recycling of nutrients [16]. In the rhizosphere, near the root–soil interface, there are high biological and chemical activities [17]. The plant roots excrete their products from photosynthesis as root exudates in the form of soluble sugars, amino acids, or secondary metabolites [18]. Therefore, the development of the microbial community in the rhizosphere is highly correlated with the root exudates of the host plant [17]. In addition, approximately 80% of terrestrial plant species form some type of mycorrhizal symbiosis, from which arbuscular mycorrhiza is the most widespread and predominant type [19]. Arbuscular mycorrhiza fungi (AMF) belonging to the phylum *Glomeromycota* [20] are obligate symbionts dependent on the host plant [21]. Plants colonized by AMF have improved resistance to environmental stresses, such as drought, cold, and pollution [22], and also are better able to overcome attacks by bacterial and fungal pathogens [23]. Moreover, they are useful in decreasing pollutants in the biosphere, including heavy metals, organic compounds, and radionuclides [24].

This study aimed to evaluate the dynamics of fungal communities, especially AMF, in the rhizosphere and roots of barley sown in soil with the addition of SS as a soil amendment at concentrations of 5 and 15 t ha^−1^. The molecular analysis of partial 18S rRNA gene was used for this evaluation, and we assumed that the presence of SS in the soil would affect the genetic diversity of fungal communities both in the rhizosphere and in the roots of barley. In addition, the effect of SS on selected parameters of barley production was determined. We supposed that the sludge would affect these parameters, and this would be more pronounced in the higher concentration of 15 t ha^−1^.

## 2 Materials and methods

### 2.1 Study description and SS used

Municipal SS used in this experiment was obtained from the wastewater treatment plant Tavos, a.s., Piešťany, Slovak Republic that collects wastewater from more than 9,000 households. This sludge was concentrated, anaerobically digested, dewatered, dried, and mechanically homogenized to a fine powder. Elemental analyses of macroelements and heavy metals (heavy metals did not exceed the limits permitted by the Directive 86/278/EEC [6]) in SS are shown in Table 1. Plants of spring barley (*Hordeum vulgare*, L.), cultivar Levan, were used in this experiment. The seeds were sown in the pots filled with 7 kg of arable soil supplemented with anaerobically digested SS at a concentration of 15.7 or 47.1 g of SS per pot, which corresponded to the application of SS at a concentration of 5 or 15 t ha^−1^, and the control plants were in soil without any SS supplement. Pots were placed in the greenhouse conditions (under natural light conditions with a photoperiod of 15 h light/9 h dark and temperature of 25°C day/18°C night) and irrigated as needed. At the same time, water-holding plates were placed under each pot to reuse the percolated water and thus to prevent the nutrients from leaching out of the pots. The soil type used was Luvi-Haplic Chernozem on loess with pH (KCl) 6.3 and a humus content of 1.77%, and the content of macroelements is shown in Table 1. The total contents of N and C were determined according to the Dumas method using a CNS analyzer (TruMac; LECO, St. Joseph, MI, USA). Other macroelements were measured by Microwave Plasma-Atomic Emission Spectroscopy (MP-AES 4100; Agilent, Santa Clara, CA, USA) after extraction from the samples by a Mehlich III solution and microwave digestion of the extracts (system Ethos 1, Milestone, Sorisole, Italy). The heavy metal content was analyzed by X-ray fluorescence spectrometry in an accredited laboratory (Department of Inorganic Analyses Laboratory, Division of Geoanalytical Laboratories, Regional Centre Spišská Nová Ves, State Geological Institute of Dionýz Štúr, Slovak Republic) with three CRMs (Certified Reference Materials) of SS with different contents of trace elements, the certificates of which were issued by the Slovak Institute of Metrology, Bratislava, Slovak Republic. Similarly, CRM GSD 12 (river sediment) was measured alongside our samples, which monitors the long-term stability of the instrument. The GSD 12 certificate was issued by the National Analysis Center for Iron & Steel, Beijing, China.

**Table 1:** Content of macroelements in soil and anaerobically digested SS and microelements/heavy metals in SS, and the conversion of heavy metal content to 1 kg of soil supplemented with SS at the concentrations of 5 and 15 t ha^−1^

Macroelements	Amount in soil (g kg^−1^)	Amount in SS (g kg^−1^)
N	0.958	35.10
P	0.097	16.663
K	0.196	2.663
C	10.30	334.0
Ca	2.940	36.395
Mg	0.280	6.444

Microelements/heavy metals	Amount in SS (g kg^−1^)	SS (g kg^−1^) soil in 5 t ha^−1^	SS (g kg^−1^) soil in 15 t ha^−1^	EU limit values* in SS (g kg^−1^)
As	0.003	0.00001	0.00002	NA
Cd	<0.001	<0.000002	<0.00001	0.020–0.040
Cr	0.036	0.00008	0.00024	NA
Cu	0.224	0.00050	0.00151	1.000–1.750
Ni	0.022	0.00005	0.00015	0.300–0.400
Pb	0.046	0.00010	0.00031	0.750–1.200
Zn	1.269	0.00285	0.00854	2.500–4.000

*Council directive 86/278/EEC of 12 June 1986 on the protection of the environment, particularly of the soil when SS is used in agriculture [6].

NA, not applicable.

### 2.2 Rhizosphere and root sampling

The rhizosphere and root samples from barley plants were collected in three stages of barley growth – GS29 in May, GS75 in June, and GS92 in July 2015 [25], and each sample was taken individually from separate pots. Three pots (three individual samples) were considered as controls with arable soil only, three pots (three individual samples) were supplemented with anaerobically digested SS with the concentration of 5 t ha^−1^, and three pots (three individual samples) with the concentration of 15 t ha^−1^. Therefore, nine samples from the rhizosphere and nine samples from the roots in each of the three developmental stages of barley were collected. Together 27 samples from the rhizosphere and 27 samples from the roots were analyzed. The rhizosphere was collected by taking the plants out of the soil, gently removing the soil residues from the roots, and then scraping the rhizosphere soil from the roots with a sterile scalpel without damaging the roots. The samples were then cooled and stored before analysis at 4°C. The remaining roots were gently rinsed in sterile water to remove any residual soil and dried at room temperature. On the sampling day, immediately after the roots had dried, they were used with the rhizosphere samples for DNA isolation.

### 2.3 DNA extraction and molecular analysis of partial 18S rRNA gene

The metagenomic DNA (mgDNA) from the rhizosphere was extracted from 0.25 g of rhizosphere samples using the PowerSoil^TM^ DNA Isolation kit (Qiagen, Hilden, Germany). The mgDNA from roots was extracted from 0.1 g of dried roots using the DNeasy Plant Mini Kit (Qiagen, Hilden, Germany). Extracted DNA from rhizosphere and roots was dissolved in 50 μL of nuclease-free water. The quantity and purity of DNA were measured spectrophotometrically with a NanoDrop-1000 Spectrophotometer (Thermo Fisher Scientific Inc., Waltham, MA, USA), and samples were diluted to the same final concentration (20 ng μL^−1^) and stored at −20°C. Molecular analysis of partial 18S rRNA gene using two conserved primer pairs NS1 with NS4 [26] and NS31 with AM1 [27] in terminal restriction fragment length polymorphism (T-RFLP) was performed according to our previous study [28]. The second primer pair is specific for AMF; however, it should be noted that it also amplifies DNA from *Ascomycota* and *Basidiomycota* to a limited extent [28,29,30].

### 2.4 Parameters of barley production

Barley plants were allowed to grow until full maturity stage (GS92). One hundred plants per treatment (control – soil without any supplement and soil supplemented with anaerobically digested SS at the concentration of 5 and 15 t ha^−1^) were used for the evaluation of barley production. Four parameters were evaluated: plant height in centimeters, the number of grains per plant, the weight of grains per plant in grams, and thousand seed weight in grams.

### 2.5 Statistical analyses

Statistically significant differences among samples were tested using analysis of variance (ANOVA) and subsequently using *post hoc* pairwise comparisons based on Fisher’s least significant difference (LSD) procedure at the 95% confidence level, using the software Statgraphics x64 (Statpoint Technologies, Inc., Warrenton, VA, USA). Diversity indices such as the Simpson index, Shannon’s diversity index, and Pielou evenness index for the evaluation of alpha diversity also were calculated according to our previous study [28]. Species richness, Simpson, and Shannon indices were also used for SHE analysis [31]. T-RFLP profiles of fungal communities in different samples were subsequently used for principal component analysis (PCA) using the scores of the first two principal components, for the scree plot, and also for neighbor-joining cluster analysis with Euclidean distance measure. Euclidean distance measure with 9999 permutations was also used for PERMANOVA analysis using scores from the first six principal components. SHE analysis, PCA, cluster analysis, and PERMANOVA were evaluated using PAST (PAleontological Statistics) software version 3.19 [32]. Graphical multifactorial ANOVA was made using the scores of principal components 1 (PC1) and 2 (PC2) from the PCA. Analysis of means plot with 95% decision limit was made using values from parameters of barley production. Multifactorial ANOVA and analysis of means plot were created using the software Statgraphics x64 (Statpoint Technologies, Inc., Warrenton, VA, USA).

## 3 Results

### 3.1 The influence of SS on the alpha diversity in the rhizosphere and roots of barley

**Figure 1:**
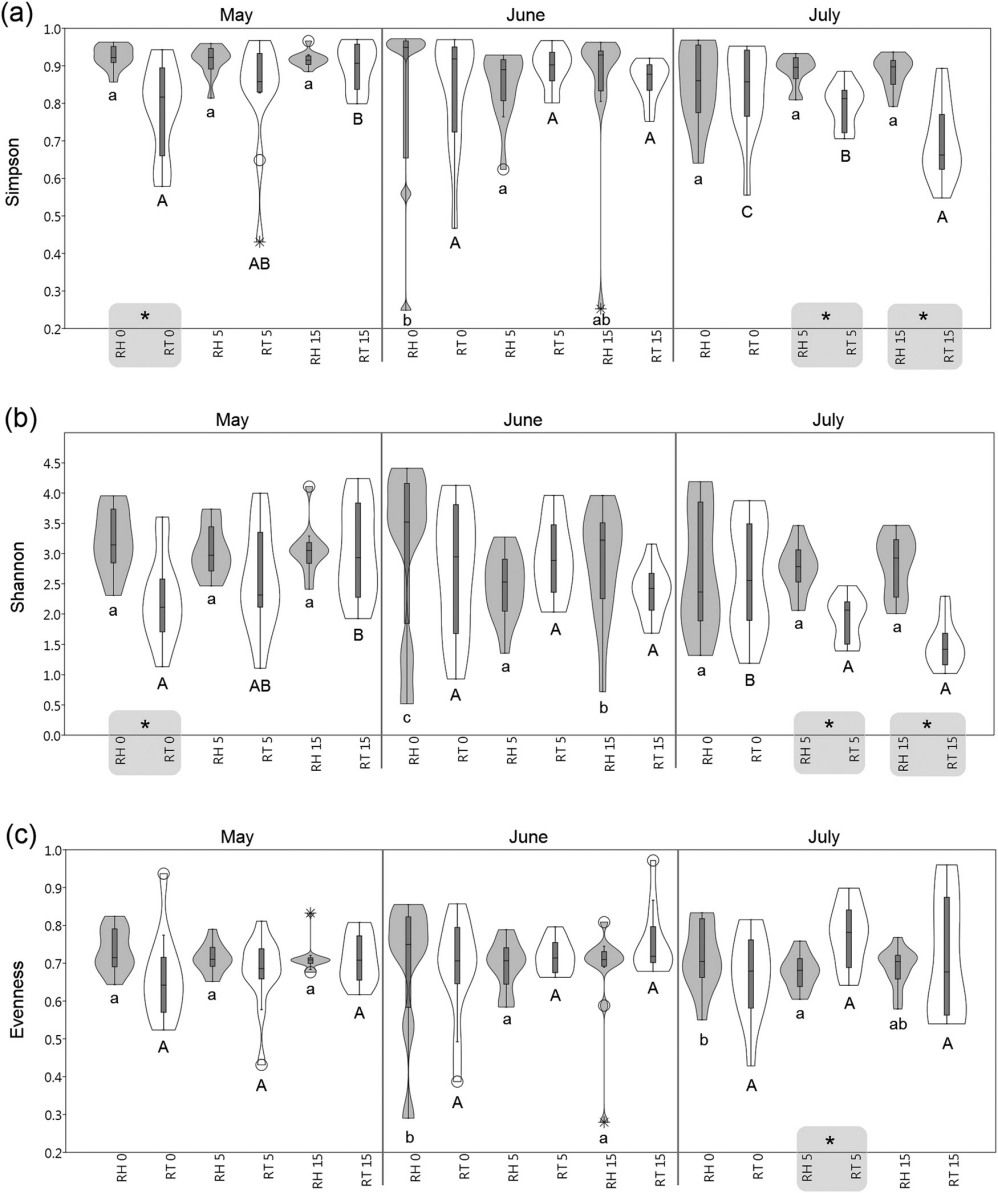
The violin and box plots of alpha diversity indices ((a) Simpson, (b) Shannon, and (c) Evenness) of fungal communities detected in the rhizosphere (RH, gray) and root (RT, white) of barley in control (RH0/RT0) and samples with SS at concentrations of 5 t ha^−1^ (RH5/RT5) and 15 t ha^−1^ (RH15/RT15). The different lowercase and capital letters denote the statistically significant differences among samples in the rhizosphere and root, respectively; values and corresponding letters indicating statistical significance go in sequence a/A < b/B < c/C; each sampling date and differences between RH and RT were evaluated separately (LSD, *α* = 0.05); *statistically significant difference between RH and RT (LSD, *α* = 0.05).

Fungal alpha diversity in the rhizosphere and roots of barley was evaluated as three diversity indices: Simpson, Shannon, and Evenness, in May, June, and July 2015 (Figure 1). The alpha diversity because of SS was otherwise manifested in the rhizosphere and otherwise in barley roots during 3 months. In the rhizosphere in May, the alpha diversity remained statistically unchanged because of the SS concentration, while in June the SS caused a statistically significant decline in Shannon and between control and 5 t ha^−1^ SS in Simpson diversity. Subsequently, in July, the Simpson and Shannon indices were statistically stable in the rhizosphere, but Evenness had a significant downward trend because of the SS concentration. On the contrary, in barley roots, the Simpson and Shannon indices in May significantly increased with increasing SS concentration. In June, diversity in the roots was not affected by the presence of SS in the soil but subsequently in July, the Simpson and Shannon diversity were statistically decreased in barley roots with SS. Evenness in the roots in all three dates was without differences between the control and SS samples. Comparing the diversity differences between rhizosphere and root, a statistically significant difference was detected in seven cases (Figure 1). In general, the mean values of Simpson and Shannon diversity were statistically higher in the rhizosphere than in the roots, but Evenness in July was statistically higher in the roots. There was a significant difference twice in May between control samples in the Simpson and Shannon diversity indices, and five times in July in all three indices but only between samples with the addition of SS to the soil.

**Figure 2:**
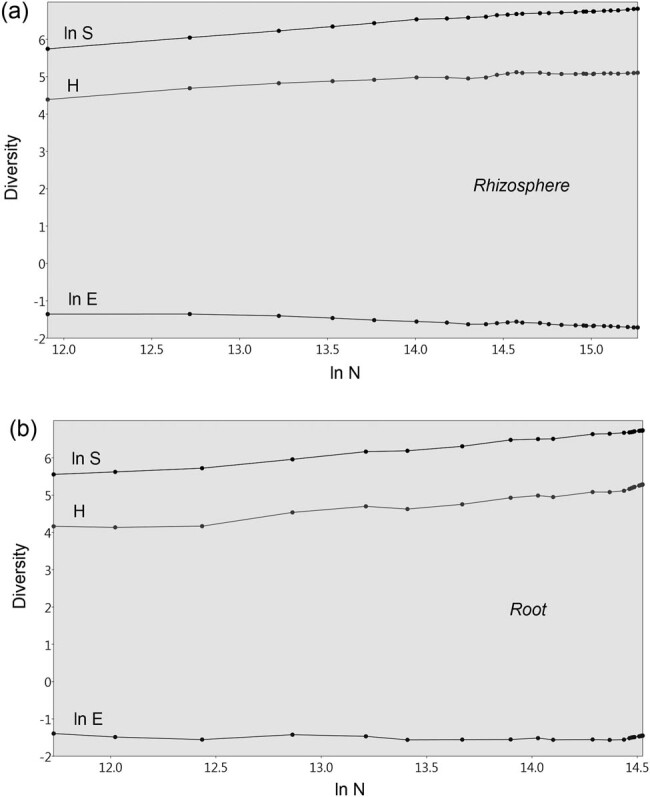
The SHE analysis of fungal diversity for rhizosphere (a) and root (b) of barley. ln *S*, natural logarithm of species richness; ln *E*, natural logarithm of evenness; *H*, Shannon diversity index; ln *N*, natural logarithm of counted individuals (in our case the height of fluorescence in individual OTUs).

SHE analysis showed that samples from the rhizosphere and roots of barley contained substantially homogeneous fungal populations despite the presence or absence of SS in the soil and sampling date. In the rhizosphere, there was a gradual increase in species and diversity with an increasing number of samples, but Evenness declined subtly (Figure 2a). In barley roots, the number of species and the diversity increased with an increasing number of samples, while the Evenness remained at the same level. Furthermore, the curves that correspond to ln *S*, *H*, and ln *E* from roots did not run as smoothly as the curves from the rhizosphere (Figure 2b).

### 3.2 The influence of SS on the composition of fungal communities in the rhizosphere and roots of barley

**Figure 3:**
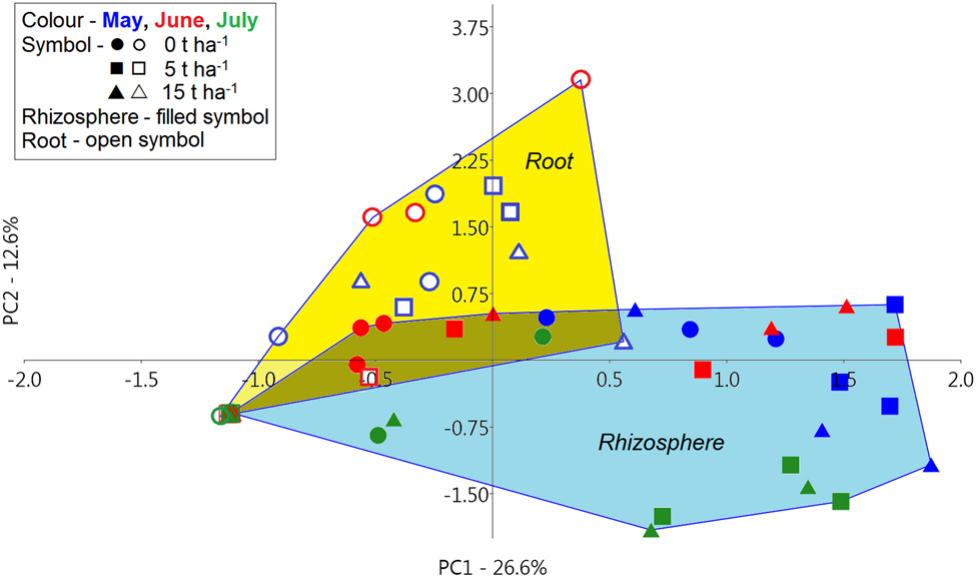
The PCA constructed from fluorescent data of fungal communities from the rhizosphere and roots of barley collected on three sampling dates in control samples and samples with SS at concentrations of 5 and 15 t ha^−1^. PCA graph explained a total of 39.2% of the variability in the data.

**Figure 4:**
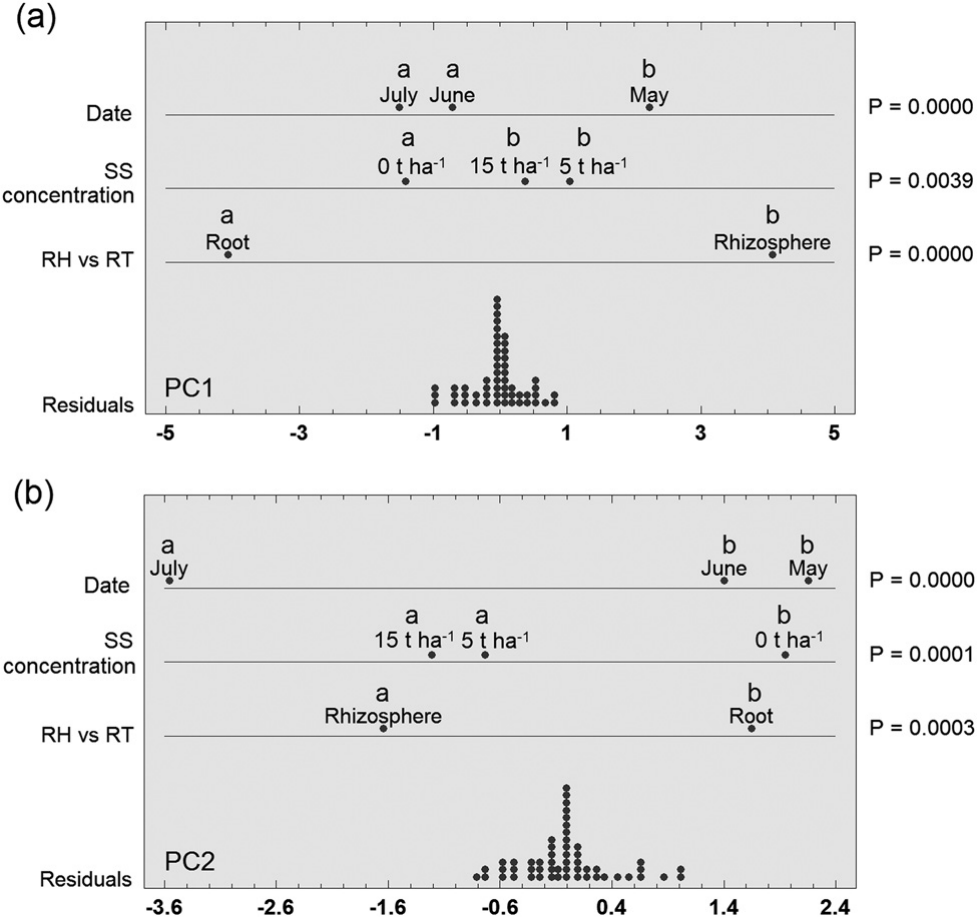
Effect of sewage sludge concentration, sample type, and sample date on the composition of fungal communities in the rhizosphere and roots of barley. Graphical multifactorial ANOVA for principal component 1 (PC1; a) and principal component 2 (PC2; b) derived from PCA in Figure 3. The different letters denote statistically significant differences among samples (LSD, *α* = 0.05). RH, rhizosphere; RT, root; SS, sewage sludge.

Based on PCA, samples from the rhizosphere and roots of barley were mostly separated from each other, although a small overlap between the two groups was recorded (Figure 3). Fungal communities from barley roots were slightly more similar based on PC1 than rhizosphere fungal communities. Furthermore, PC1 divided only four samples from roots and root samples were mostly distributed in the left side from this first axis. As the similarity or difference of the fungal communities could not be evidently demonstrated from the PCA plot between the rhizosphere and root, or between the controls and SS concentrations, or among the three sampling dates, ANOVA using these three factors (sample type, SS concentration, and date) was performed using the scores for PC1 and PC2 (Figure 4). It is obvious from the multifactorial ANOVA that PC1 and PC2 significantly divided rhizosphere fungal communities from root fungal communities as well as fungal communities in control samples from fungal communities in soil with SS addition. Regarding the sampling date, PC1 statistically separated May from June and July, but PC2 separated May and June from July.

**Figure 5:**
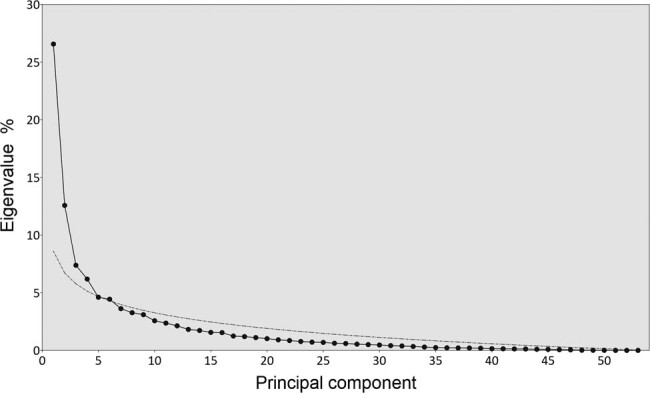
Influence of SS concentration on the composition of fungal communities in the rhizosphere and roots of barley. Scree plot indicating the percentage of eigenvalues for all 53 principal components (dotted line) derived from PCA from Figure 3. Eigenvalues under dashed line may represent nonsignificant components [33]. The first six significant components explained a total of 61.7% of the variability.

The previous PCA resulted in 53 principal components expressing a specific degree of variability. PC1 determined the greatest variability, which decreased as the number of components increased (Figure 5). However, not all these components were significant. Based on the scree plot, it could be assessed that the first six components were significant for a thorough evaluation of the PCA. PC1–PC6 explained a total of 61.7% of the variability and are sufficient for this evaluation. Therefore, scores of the first six significant components were used to determine the impact of two factors (rhizosphere vs root, SS concentration) on the genetic diversity of fungal communities using two-way PERMANOVA (Table 2). It was confirmed that both evaluated factors had a significant impact on the genetic diversity of fungal communities. The date as a factor was evaluated separately using one-way PERMANOVA and also, in this case, a statistically significant difference was confirmed (*P* = 0.0001, data not shown).

**Table 2:** Results of the two-way PERMANOVA calculated from the obtained data using the first six significant component scores derived from the PCA in Figure 3 and the scree plot in Figure 5

Similarity index	Euclidean distance
Permutation *N*	9999
	*P*-value
RH vs RT	0.0001
SS concentration	0.0248
Interaction	0.2095

RH, rhizosphere; RT, root; SS, sewage sludge.

**Figure 6:**
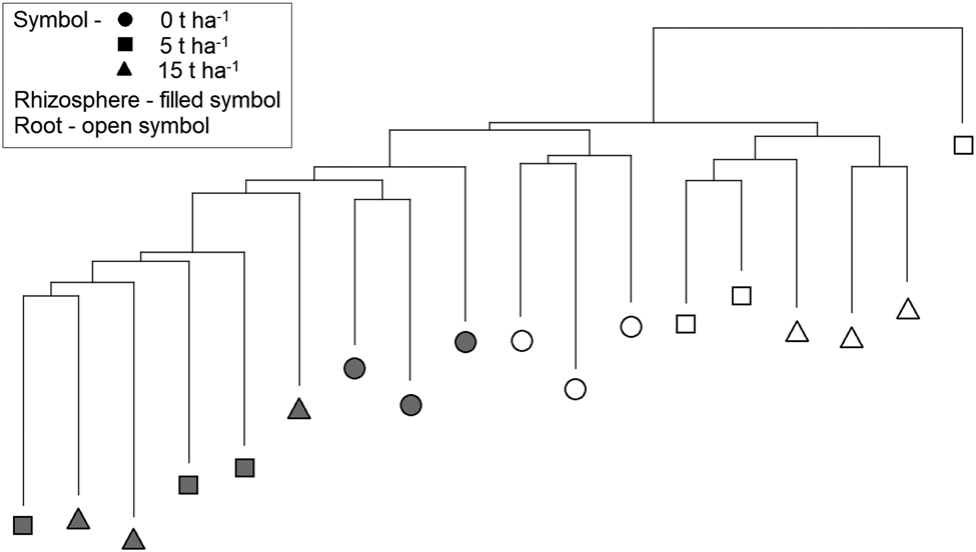
Neighbor-joining cluster analysis with Euclidean distance measure constructed from fluorescent data of fungal communities from the rhizosphere and roots of barley collected in control samples and samples with SS at concentrations of 5 and 15 t ha^−1^.

Further analysis was done to determine the effect of SS on the fungal genetic diversity in the rhizosphere and barley roots, but the sampling date was removed as a factor. Cluster analysis separated rhizosphere samples from roots and also control samples from samples with SS addition into the soil (Figure 6). However, the fungal communities were not separated based on the amount of SS in the soil. Fungal communities in control samples from barley roots formed a separate cluster, while the three rhizosphere control samples did not form a separate cluster. One control sample from the rhizosphere was on a separate cluster, although it was located near the other controls.

### 3.3 The influence of SS on the barley production parameters

**Figure 7:**
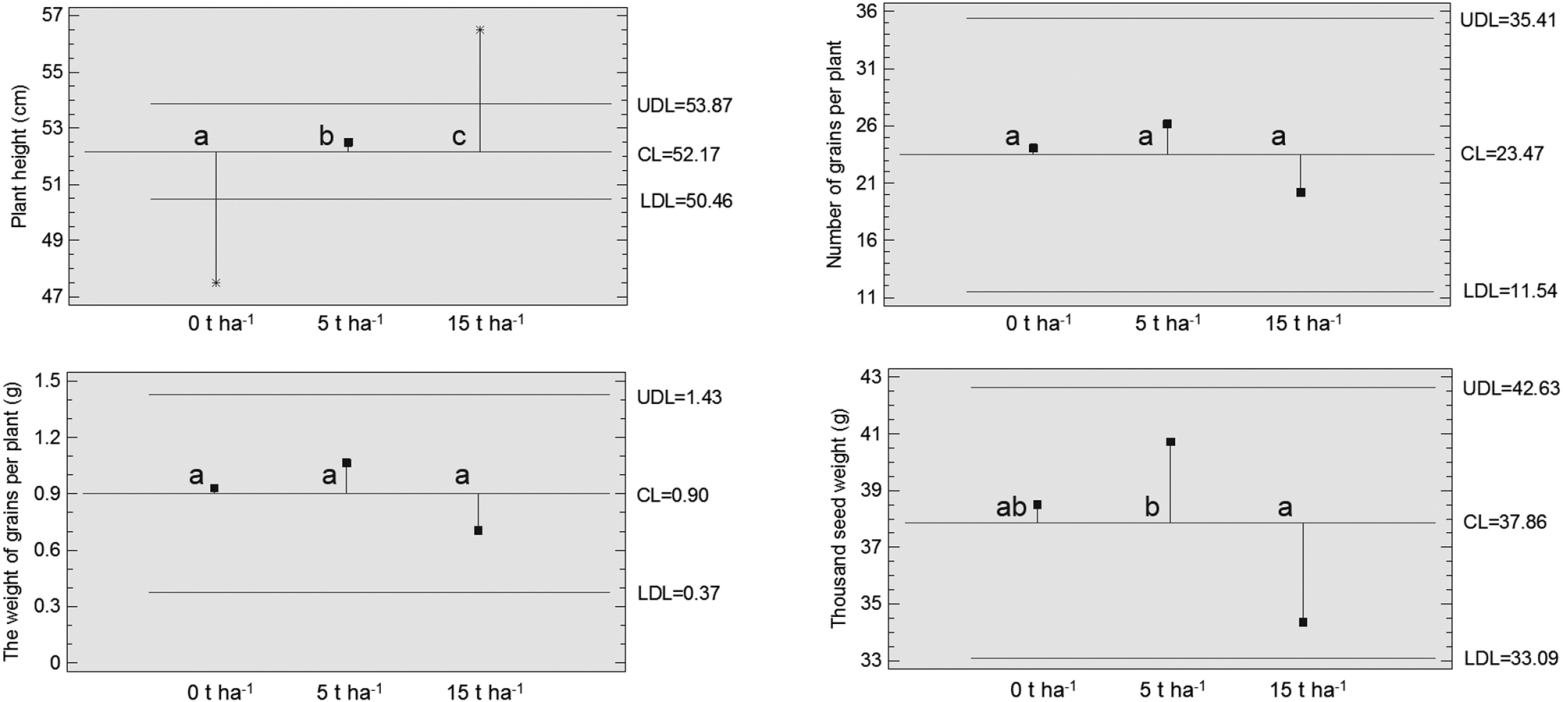
Analysis of means plot with 95% decision limit for the effect of SS concentration (0, 5, and 15 t ha^−1^) on parameters of barley production including plant height, number and weight of grains per plant, and thousand seed weight. The different letters denote statistically significant differences among samples (LSD, *α* = 0.05). UDL, upper decision limit; CL, central limit; LDL, lower decision limit.

Barley production was evaluated by four parameters: plant height, number of grains per plant, the weight of grains per plant, and thousand seed weight (Figure 7). Plant height was significantly affected by SS concentration and plants were clearly the largest at the concentration of 15 t ha^−1^. The number of grains per plant and the weight of grains per plant were not statistically affected by the SS concentration. However, there was a trend that these two parameters decreased at the concentration of 15 t ha^−1^ compared to the control samples. The last parameter, thousand seed weight, was influenced by the presence of SS in the soil at the concentration of 5 t ha^−1^. Interestingly, this parameter at 5 t ha^−1^ was not statistically different from the control samples but differed from the samples at the 15 t ha^−1^ concentration. Besides, the value of this parameter at 15 t ha^−1^ was the lowest and well below the overall average (central limit, Figure 7).

## 4 Discussion

Using biological approaches to determine the ecological effects of land application of municipal SS has been preferred widely for decades [4,28]. Alpha diversity [34] indicates the diversity that exists within a sample/location, and it is measured by the number of species or the richness of species but other diversity indices are often used, such as Shannon and Simpson [35]. The results of the present study indicate that the application of sludge to the soil influenced the alpha diversity of fungal communities in the rhizosphere and barley roots differently. The Simpson and Shannon indices from the rhizosphere of barley collected on three sampling dates had the following significant trend from control to sludge samples: without change (May) – downward trend (June) – without change (July). In the roots of barley, these indices were as follows: upward trend (May) – without change (June) – downward trend (July). These figures evidence that in the final tillering stage (GS29) [25] in May, the root fungal diversity was significantly affected by the presence of SS in the soil, which caused an increase in the diversity in barley roots compared to the control. Subsequently, in the barley late milk development stage (GS75) [25] in June, the SS significantly decreased fungal diversity in the rhizosphere but was without a change in the roots. On the contrary, in the barley final ripening stage (GS92) [25] in July, the SS significantly decreased the root fungal alpha diversity compared to the control. Similarly, the impact of SS spreading under field conditions on AMF species in the soil and within root systems of *Medicago truncatula* was studied by Jacquot-Plumey et al. [36], who detected the different impact of SS on AMF diversity between the soil and the root systems of *M. truncatula*. While in the soil the effect of composted SS on AMF diversity was manifested by its increase, the diversity of AMF in the roots was lower in the presence of SS and significantly higher in the control. In addition to SS such as in our case, high ash applications also enhanced mycorrhizal status in barley compared to the control and triple superphosphate application [37]. Figueiredo et al. [38] and Yusif et al. [39] came to a similar finding when they used SS biochar, which increased the mycorrhizal colonization of corn roots in relation to the control [38] or enhanced root colonization in tomato genotypes [39]. Even, increasing the heavy metal content in the soil, which often occurs following SS application, may increase mycorrhizal colonization of the plant roots [40,41].

Shannon information function (*H*), species richness (*S*), and Evenness (*E*) also can be used in ecological studies for SHE analysis, which examines the relationship among *S*, *H*, and *E* in the samples [31]. In this case, *H* = ln *S* + ln *E* and this decomposition formula expresses that relationship in one plot with three variables/curves that are plotted against the abundance (*N*) of the sample [42,43,44]. At the same time, these variables form linear trends on a log scale, and when *N* accumulates with each sample, *S* usually increases [42]. There are several ways in which the curves for *H* and ln *E* can proceed (increasing, constant, or decreasing trends) but simultaneously, any departures from linear trends indicate a mixture of communities [42,45]. In this study, SHE analysis showed that rhizosphere fungal communities formed more homogenous communities than those in the roots. On the contrary, it should be noted that although the curves for ln *S*, *H*, and ln *E* from the roots did not run as smoothly as those from the rhizosphere, they still maintained a linear trend on a log scale without departures. These results suggest that, from an ecological perspective, fungal communities from roots and the rhizosphere formed homogeneous communities even in the presence of SS in the soil at two different concentrations.

The PCA and cluster analysis revealed that fungal communities were strongly influenced by the SS addition to the soil, sample type, and the sampling date. Generally, SS is composed of organic compounds, micro- and macronutrients, non-essential trace metals, organic micropollutants, and microorganisms [3]. Therefore, SS added to the soil can modify soil structure, moisture, porosity, humus content, pH, electrical conductivity, or cation exchange capacity. [4,46,47,48]. Besides, SS also changes soil microbial communities [4,49,50], which was the case with our study as well. In particular, several studies have focused on monitoring changes in the dynamics of the microbial community because of the addition of sludge containing higher concentrations of potentially toxic elements/heavy metals such as Cu, Zn, Cd, and others [51,52,53,54,55,56,57,58]. Interestingly, Gomes et al. [54] found that soil fungal communities were influenced by the quality and amount of SS soil application but not by Cd and Zn at higher concentrations. Similar results were also obtained by Anderson et al. [53] who observed that sludge type had the greatest effect on the soil fungal communities rather than SS rich in Cd, Cu, or Zn. Likewise, the results of Lloret et al. [59] were interesting, in a comparative study of the effect of SS addition on two different sludge stabilization processes on soil bacterial, archaeal, and fungal communities, while heavy metal concentrations were below the limits set by the EU. From various fungal communities, only the relative abundance of *Glomeromycota* was significantly increased in all amendments. Our current results regarding the addition of SS to the soil and the observed changes in the fungal communities in the rhizosphere and roots of barley are in agreement with these abovementioned studies. On the contrary, we previously observed different results in our parallel experiment with the same concentrations of SS added to the soil and monitoring the dynamics of the fungal communities in the *Arundo donax* rhizosphere [28]. In this published paper, we used the same methodology and similar statistical evaluation, but the SS did not cause a shift in the overall rhizosphere fungal communities through PCA and analysis of similarities (ANOSIM) analysis. Only sequencing of 18S rDNA showed that more various fungal taxa were detected in the sample with SS than in the control. We assume that these different results between our two experiments were mainly because of the plant species, i.e., barley (*Hordeum vulgare*, L.) vs *A. donax*. This assumption reflects the fact that plant roots release a wide range of chemicals in the form of root exudates into the soil, which subsequently determine the plant–microbe interaction in the rhizosphere [60]. Furthermore, the quantity and quality of root exudates depend on the plant species, plant developmental stage, and various biotic and abiotic factors. Together, all these factors play a pivotal role in determining specifically the strength and type of microorganisms present in the rhizosphere [18,61].

Currently, there is an increasing interest worldwide in the use of SS in agriculture because of the possibility of recycling valuable components such as organic matter, N, P, and other plant nutrients [4]. A minor part of the current study was monitoring the effect of SS as a soil amendment on some selected parameters of barley production. The plant height increased with increasing concentration of SS. In general, the addition of SS to agricultural soil increases the growth and production of cultivated plants [3]. SS also increased yield parameters of barley and also enhanced protein content compared to unfertilized soil [62] as well as growth and N uptake [63]. Kępka et al. [64] observed that spring barley yield was mainly influenced by N coming from the SS applied. At the same time, they mentioned that their applied SS at a concentration of 5.34 t ha^−1^ dry matter met the nutrient requirements of N by spring barley, and along with the applied concentration of SS, more than 118 kg N ha^−1^ was introduced. Antolín et al. [47] investigated the effects of SS on the relationships between barley physiology and some soil properties during a 3-year period. They detected that repeated yearly application of SS to barley crops resulted in increased grain and dry matter yields and leaf protein concentrations. Application of SS also improved soil chemical, microbiological, and biochemical properties, which were reflected in an increase in barley yield. However, they detected a significant increase in heavy metal concentrations in barley grains. Similarly, Fernandez et al. [65] evaluated the effects of composted and thermally dried SSs with different frequencies (single or yearly applications) and at two application concentrations (20 and 80 t ha^−1^) on the yield of barley during a 3-year period. They observed that in the cumulative experiment high concentrations of both SSs caused a significant decrease in crop yield, but in contrast, cumulative applications of both types of SS at low concentrations showed, in general, better barley yield parameters. Moreover, Eid et al. [66] studied the impact of different SS concentrations (10, 20, 30, 40, and 50 g kg^−1^) on soil properties and barley yield. The best results with enhanced barley growth were achieved at the 40 g kg^−1^ concentration of SS, while all barley growth parameters were decreased at the concentration of 50 g kg^−1^. We observed a similar effect of different SS concentrations on one of the measured barley parameters – thousand seed weight. This parameter was significantly increased at the SS concentration of 5 t ha^−1^ but significantly decreased at 15 t ha^−1^. Eid et al. [66] explained these findings result from the fact that the high concentration of SS is composed of high levels of some heavy metals that may affect plant metabolic activities as well as plant growth [67,68,69].

## 5 Conclusions

The present study showed that short-term application of SS to the soil at the concentrations of 5 and 15 t ha^−1^ affected fungal communities, especially AMF in the rhizosphere and roots of barley. Both concentrations of SS affected these fungal communities to a comparable extent without a significant difference between them, while the yield of barley was positively affected by SS at the concentration of 5 t ha^−1^. However, other similar studies need to be conducted to understand the interactions and feedback between the application of SS to the agricultural soil and the structure and function of microbial communities in the rhizosphere and plant roots to develop an optimal and safe use of SS in agriculture.
